# Morphological and molecular characterisation of *Myxobolus pronini* n. sp. (Myxozoa: Myxobolidae) from the abdominal cavity and visceral serous membranes of the gibel carp *Carassius auratus gibelio* (Bloch) in Russia and China

**DOI:** 10.1186/s13071-016-1836-3

**Published:** 2016-10-25

**Authors:** Xin-Hua Liu, Marina-D Batueva, Yuan-Li Zhao, Jin-Yong Zhang, Qian-Qian Zhang, Tong-Tong Li, Ai-Hua Li

**Affiliations:** 1 Fish Diseases Laboratory, State Key Laboratory of Freshwater Ecology and Biotechnology, Institute of Hydrobiology, Chinese Academy of Science, 430072 Wuhan, China; 2 Institute of General and Experimental Biology of Siberian Branch RAS, Ulan-Ude, Russia; 3 University of Chinese Academy of Science, Beijing, 10049 China; 4 Chengdu Institute of Biology, Chinese Academy of Sciences, 610041 Chengdu, China

**Keywords:** *Myxobolus pronini* n. sp, Abdominal cavity, Serous membranes, Geographical variation, Gibel carp, *Carassius auratus gibelio*, Russia, China

## Abstract

**Background:**

Myxozoa is a well-known economically and ecologically important group of metazoan parasites, phylogenetically related to Cnidaria. High diversity of myxosporeans has been recorded in Russia and China; however, most of the species were solely morphologically characterised. Here, we identified a new gibel carp-infecting *Myxobolus* species and morphologically and molecularly compared the Russian and Chinese isolates of this new myxosporean.

**Results:**

*Myxobolus pronini* n. sp. was found free in the abdominal cavity of *Carassius auratus gibelio* (Bloch, 1782) in Lake Baikal watershed, Russia, and embedded in the visceral serous membranes of the same fish species in Lake Taibai, Hubei province, China. The morphometric data of the plasmodia and mature spores exhibited some differences between the Russian and Chinese isolates, but SSU rDNA sequences indicated that these two geographical isolates are conspecific. The mature spores from the two locations are obovate in frontal view, with wider anterior than posterior end and lemon-shaped in sutural view. Spores of the Russian isolate were 14.3–16.2 (mean 15.1 ± 0.2) μm long, 9.6–10.8 (10.1 ± 0.1) μm wide and 6.4–7.4 (6.7 ± 0.15) μm thick; those of the Chinese isolate were 13.8–15.6 (14.7 ± 0.24) μm long, 9.6–13.3 (9.6 ± 0.65) μm wide and 6.2–7.2 (6.6 ± 0.16) μm thick. The newly-generated rDNA sequences (including SSU rDNA, ITS and LSU rDNA) from the two isolates represented some variations within the intraspecific range. Homology search by BLAST showed that the newly obtained rDNA sequences do not match any sequences available on GenBank. Phylogenetic analysis based on the aligned partial SSU rDNA sequences indicated that this novel species clustered with several gibel carp-infecting *Myxobolus* spp. with round anterior end of spores. Additionally, phylogenetic analysis based on all obtained ITS sequences showed that distinct genetic geographical differentiation occurred for this new parasite.

**Conclusions:**

*Myxobolus pronini* n. sp. is described by integrating morphological, ecological and molecular evidence. Two geographical isolates of this species showed some morphological and genetic differences but within the intraspecific range of variation.

## Background

Myxozoa Grassé, 1970 is an economically and ecologically important group of metazoan parasites. Although more than 2,200 species have been reported from aquatic invertebrates, fishes, reptiles, birds and mammals, including humans worldwide [1], high hidden diversity of this group of parasites is widely acknowledged [2, 3]. For instance, no malacosporean has been reported in China for no studies concerned this group of myxozoans. Among reported myxozoans, some were described and identified solely upon spore morphology [4–6]. These described species with insufficient data should be validated or revised by supplementing with detailed taxonomic characteristics. Currently, an integrated approach combining spore morphology, exact location of sporulation, tissue- and host-specificity and molecular characteristics has been widely recommended and accepted for identification of novel or cryptic myxosprean species and for discrimination of species with morphological similarity [7]. The genus *Myxobolus*, comprising more than 900 species is the most speciose within the phylum Myxozoa [8, 9]. Among them, about 500 *Myxobolus* species have been described or recorded from Russia and China [10, 11], although many species were possibly misidentified [6]. As a part of an ongoing joint project applying modern taxonomic features to approve the validity of the incompletely described existing taxa and uncover the actual species diversity of fish myxosporeans in Russia and China, a new species, designated here as *Myxobolus pronini* n. sp. from the abdominal cavity and the visceral serous membranes of the gibel carp *Carassius auratus gibelio* (Bloch, 1782) inhabiting in the watershed of Lake Baikal in Russia and Yangtze River in China, is morphologically and molecularly characterised in this paper.

## Methods

### Sample collection and morphological identification

Twelve gibel carp, 9.5–19.7 cm in body length, were captured from a pond near Barguzin River (53°69'N, 109°80'E), a tributary of Lake Baikal, by gill net in June of 2015. Twenty gibel carp, 16.5 to 31.3 cm in body length, were obtained from Lake Taibai (29°58'18"N, 115°23'9"E), located at the middle and lower reaches of Yangtze River, by trawl net in November of 2015. Russian and Chinese fish samples were frozen and transported in iceboxes to the laboratory in Ulan-Ude and Wuhan, respectively. Complete parasitological examination was performed. After necropsy, visual examination of the gills, muscle, liver, intestine, spleen, heart and gallbladder was performed to find the suspected plasmodia of myxosporean parasites by naked eye and further examined by preparing wet mounts of all inspected organs with stereomicroscopy and phase contrast microscopy. Frozen isolated plasmodia of the Russian isolate were transported to the laboratory of Institute of Hydrobiology, Chinese Academy of Sciences for further morphological observation and molecular characterisation.

Morphological and morphometric data of randomly selected 50 normal mature spores were obtained and measured from smear preparations of ruptured by needle plasmodia according to Lom & Arthur [12] using an Olympus BX53 microscope equipped with an ocular micrometer. Thawed spores of the two geographical isolates were photographed with the Zeiss Axiplan 2 Image and Axiophot 2. Line drawings were made based on the photographs with the aid of Adobe Photoshop CS (Adobe Systems, San Jose, CA, USA). The presence of iodinophilous vacuole and mucous envelope of the spores was checked by mixing a small drop of Lugol’s solution and black Indian ink with water suspension of fresh spores. All measurements are given in micrometres and are presented as the range followed by the mean ± standard deviation (SD) in parentheses, unless otherwise indicated.

### Genomic DNA isolation and sequencing

Preserved isolated plasmodia or myxospores in 95 % ethanol were centrifuged at 4,000× *g* for 10 min and washed two times with distilled water to remove the ethanol remnants. Genomic DNA was isolated from the obtained pellet using the Qiagen DNeasy Blood & Tissue Kit (Qiagen, Düsseldorf, Germany), following the manufacturer’s recommended protocol for animal tissue. gDNA concentration was determined using a NanoDrop2000 spectrophotometer (Thermo Scientific) at 260 nm. Ribosomal DNA fragments, including small subunit ribosomal DNA (SSU rDNA), internal transcribed spacer (ITS) and large subunit ribosomal DNA (LSU rDNA) were amplified using the primers listed in Table 1. PCR was carried out in a 25 μl reaction mixture which comprised 30 ng template DNA, 1× PCR mixture (CWbiotech, Beijing, China) and 10 pmol of each primer. The partial SSU rDNA was amplified using the primers MyxospecF-18R [13, 14] and the PCR cycle consisted of an initial denaturation step at 95 °C for 4 min, followed by 35 cycles at 95 °C for 1 min, 48 °C for 1 min, 72 °C for 2 min, and a final extension at 72 °C for 10 min.

**Table 1:** Primers used for PCR amplification or sequencing of SSU rDNA, ITS and LSU rDNA of *Myxobolus pronini* n. sp

Primer	Sequence	Reference
MyxospecF	TTCTGCCCTATCAACTTGTTG	[15]
18R	CTACGGAAACCTTGTTACG	[13]
18R-V	CGTAACAAGGTTTCCGTAG	[13]
Myxo28S1F-V	CACTTCACTCGCAGTTACT	[14]
NLF160	ACCTCCACTCAGGCAAGATTA	[16]
NLR1694	TCTYAGGAYCGACTNAC	[16]
NLF1050	AATCGAACCATCTAGTAGCTGG	[17]
NLR3284	TTCTGACTTAGAGGCGTTCAG	[17]

The complete ITS was amplified using the primer pair 18R-V/Myxo28S1F-V [13, 15] and the PCR cycle comprised by an initial denaturation step at 94 °C for 5 min, followed by 35 cycles at 94 °C for 50 s, 54 °C for 50 s, 72 °C for 2 min and a final extension at 72 °C for 10 min. The complete LSU rDNA sequence was obtained by assembling two overlapping parts. The first part of LSU was obtained using the NLF160/NLR1694 primer pair [16] and the second part using the NLF1050-NLR3284 primer pair [17]. PCR cycling parameters for LSU rDNA amplification were 95 °C for 3 min, then 30 cycles of 95 °C for 1 min, 48 °C for 1 min, 72 °C for 2 min, followed by 10 min incubation at 72 °C. PCR products were separated by agarose gel electrophoresis, purified with a PCR purification kit (CWBiotech, Beijing, China) and then cloned into PMD-18 T vector system (Takara, Tokyo, Japan). Then positive clones were selected and sequenced with the ABI BigDye Terminator v3.1 Cycle Sequencing Kit with an ABI 3100 Genetic Analyzer. Potential intraspecific geographical variations of the ITS were tested by sequencing of three clones per isolate.

### Phylogenetic analysis

All sequences were assembled in BioEdit [18] and the consensus sequences obtained were determined as myxozoan by a GenBank BLAST search. To explore the phylogenetic relationships of the present species with the existing myxobolids, 37 SSU rDNA sequences of selected myxosporean species were aligned with Clustal X version 1.8 [19] by default setting. All newly-generated ITS sequences of *Myxobolus pronini* n. sp. and several cyprinid-infecting myxosporean species were also aligned to explore the possible geographical variation of this species. The alignment was corrected manually using the alignment editor of the software MEGA 6.0 [20]. Pairwise sequence distances and similarity of clones of the two geographical isolates based on ITS were calculated in MEGA 6.0 [20]. Phylogenetic analysis was performed using maximum likelihood (ML) analysis in PhyML 3.0 [21] and Bayesian analysis in MrBayes [22]. Optimal evolutionary model for ML and Bayesian analysis was determined using Modeltest 3.7 [23] which identified the best evolutionary model for SSU rDNA and ITS dataset were the general time reversible model (GTR + I + G) and HKY + I, respectively, judging by the Akaike information criterion. Two independent runs were conducted with four chains for a million generations for Bayesian analysis. Phylogenetic trees were sampled every 100 generations. The first 25 % of the samples were discarded from the cold chain (burninfrac = 0.25). Bootstrap confidence values were calculated with 100 pseudoreplicates for ML. *Ceratonova shasta* Atkinson, Foott & Bartholomew, 2014 (AF001579) and *Myxobolus cerebralis* Hofer, 1903 (AY479922) were used as the outgroup taxa for SSU rDNA and ITS dataset, respectively. Trees were initially examined in TreeView X [24] and then edited and annotated in Adobe Illustrator (Adobe Systems Inc.).

## Results

**Fig. 1:**
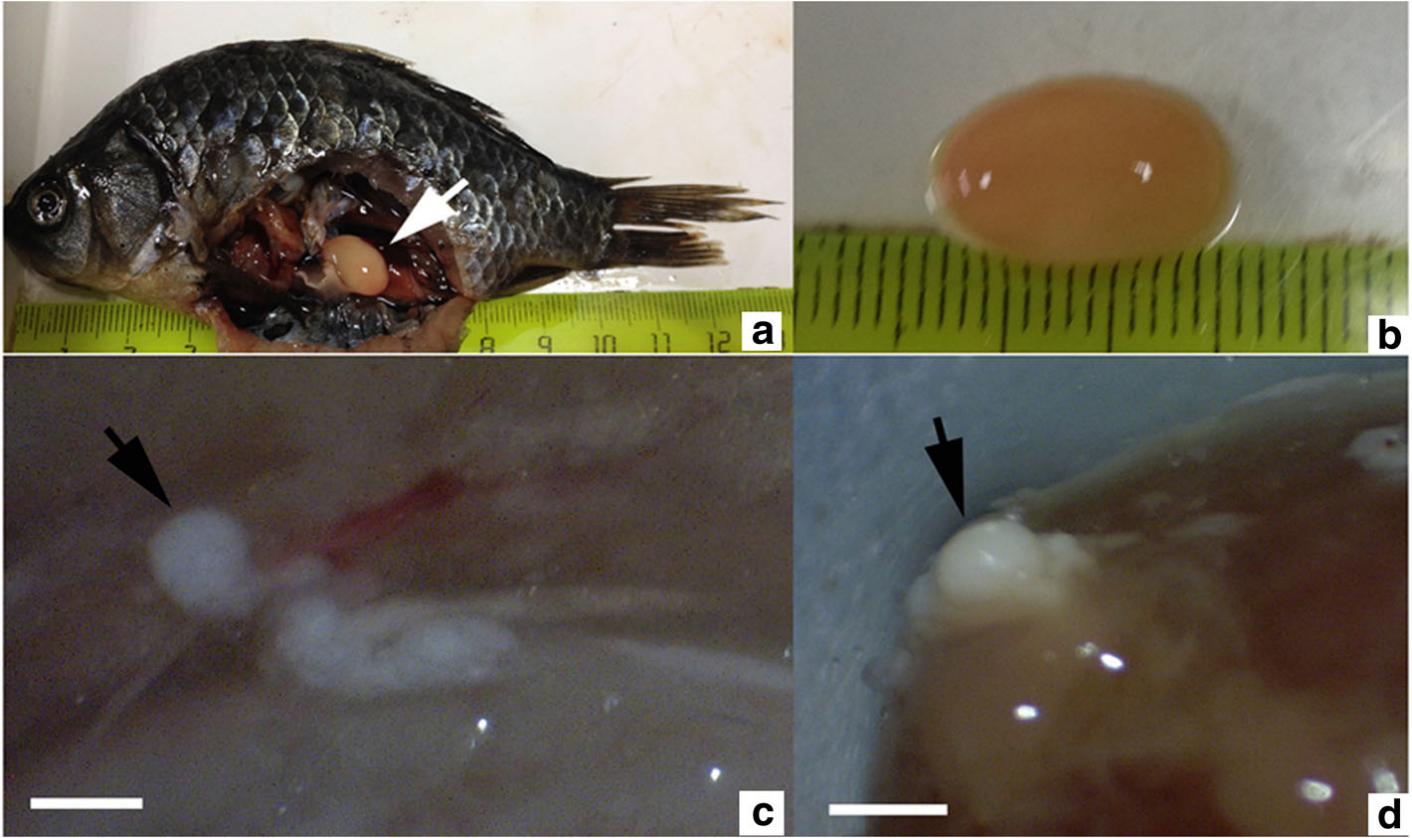
The plasmodium of *Myxobolus pronini* n. sp. **a**, **b** Large yellowish plasmodium (*arrow*) free in the abdominal cavity of *Carassius auratu*s *gibelio* sampled from a pond near Lake Baikal, Russia; **c**, **d** A single small whitish plasmodium (*arrow*) in the serous membrane of the liver of *C. auratu*s *gibelio* sampled from Lake Taibai of Yangtze River watershed, China. *Scale-bars*: 1 mm

No clinical symptoms were observed for the infected fish from China, but slightly swelling abdomen was observed in infected fish from Russia. After dissection, a single big oval and yellowish plasmodium, measuring 1.4 × 0.93 cm was found free in the abdominal cavity of each infected host from Russia, while several small oval and whitish plasmodia, measuring 0.85 × 0.65 mm was found to protrude out of the surface of visceral serous membranes of each infected host from China (Fig. 1). The prevalence of this parasite was 25 % (3/12) and 5 % (1/20) for the Russian and Chinese isolates, respectively. Plasmodium wall consisted of a layer of collagen fibres in both isolates and no host inflammatory response was observed.

**Family Myxobolidae Thélohan, 1892**

**Genus** ***Myxobolus*** **Bütschli, 1882**

***Myxobolus pronini*** **n. sp.**

***Type-host*** **:** Gibel carp *Carassius auratus gibelio* Bloch, 1782 (Cypriniformes: Cyprinidae).

***Type-locality*** **:** Pond near Barguzin River (53°69'N, 109°80'E), Lake Baikal watershed in Russia.

***Other localities*** **:** Lake Taibai in Hubei Province, China.

***Site in host*** **:** Abdominal cavity and visceral serous membranes.

***Prevalence*** **:** 25 % (3/12) in Russia and 5 % (1/20) in China.

***Type-material*** **:** Hapantotype specimens of spores in glycerine gelly, phototypes, fixed in 10 % formalin and 95 % ethanol were deposited in the Laboratory of Fish Diseases, Institute of Hydrobiology, Chinese Academy of Science [collection numbers MP20150719 (Russian isolate) and MP20151110 (Chinese isolate)].

***Representative DNA sequences*** **:** Representative rDNA sequences were deposited in the GenBank database under the accession numbers KU524889–KU524890 (SSU); KU524891–KU524892 (LSU); and KU524893–KU524898 (ITS).

***ZooBank registration*** **:** To comply with the regulations set out in article 8.5 of the amended 2012 version of the *International Code of Zoological Nomenclature* (ICZN), details of the new species have been submitted to ZooBank. The Life Science Identifier (LSID) of the article is urn:lsid:zoobank.org:pub:A7B0E5DD-69BE-4C62-B1F6-354FB84689BA. The LSID for the new name *Myxobolus pronini* is urn:lsid:zoobank.org:act:6774338 F-6897-410A-8CB1-EB6211CF647E.

***Etymology*** **:** The species is named after Professor Nikolai Martemyanovich Pronin (Institute of General and Experimental Biology of Siberian Branch, Russian Academy of Sciences) in recognition of his significant contribution to the knowledge of fish parasites in Siberia, Russia.

### Description (Figs. 1, 2, 3, 4 and 5)

**Fig. 2:**
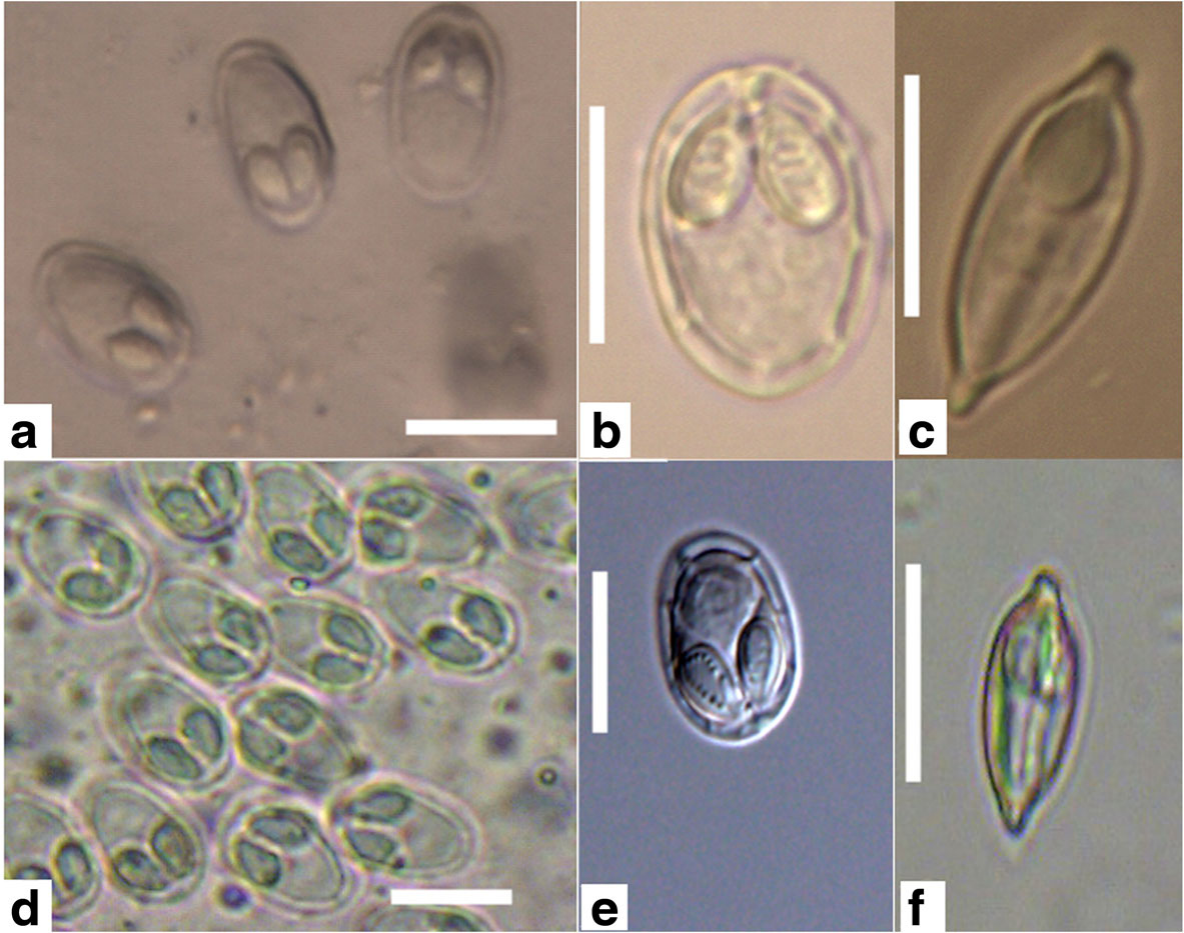
Myxospores of *Myxobolus pronini* n. sp. under light microscopy. **a**-**c** The Russian isolate. **d**-**f** The Chinese isolate. **a**, **b**, **d**, **e**, frontal view; **c**, **f**, sutural view; *Scale-bars*: 10 μm

**Fig. 3:**
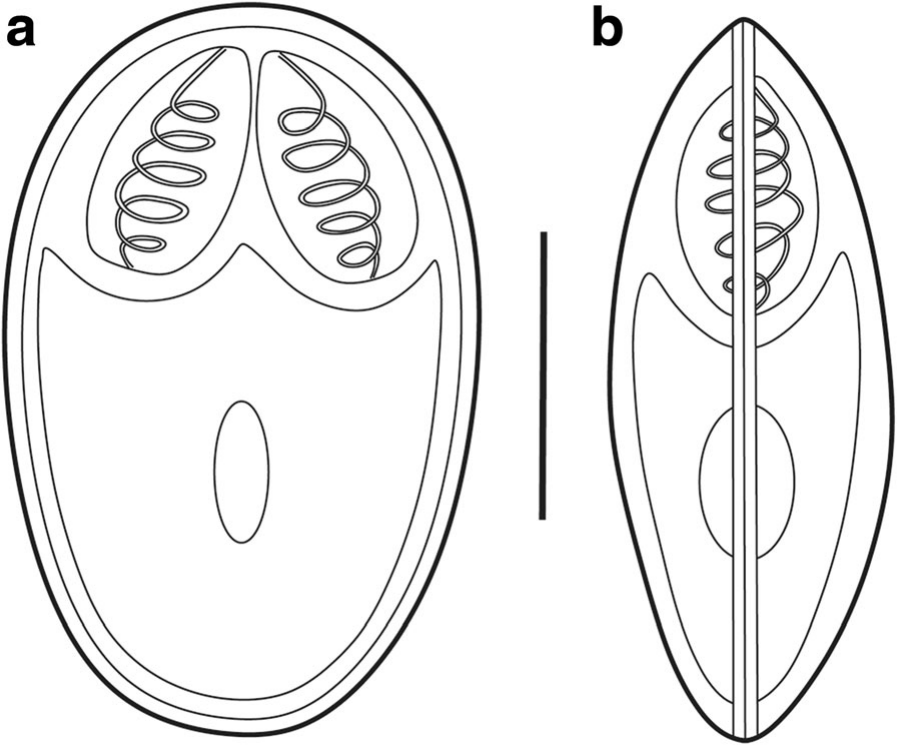
Schematic drawings of myxospores of *Myxobolus pronini* n. sp. **a** Frontal view. **b** Sutural view. *Scale-bar*: 5 μm

**Fig. 4:**
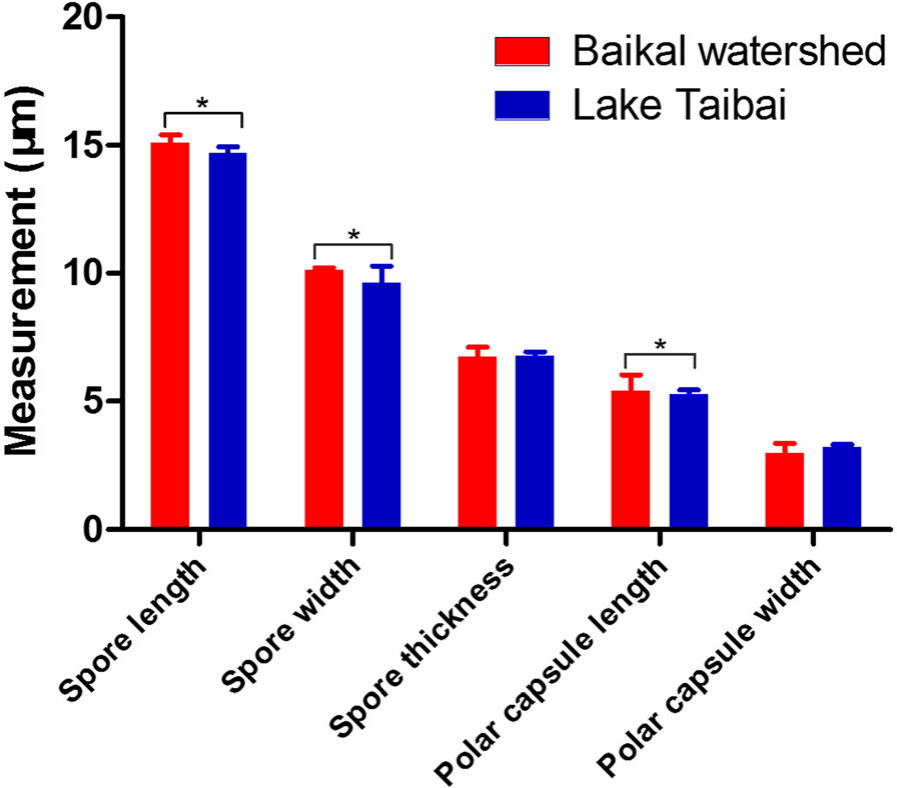
Morphometric differences between the Russian and Chinese isolates of *Myxobolus pronini* n. sp. Asterisks indicate significant differences

**Fig. 5:**
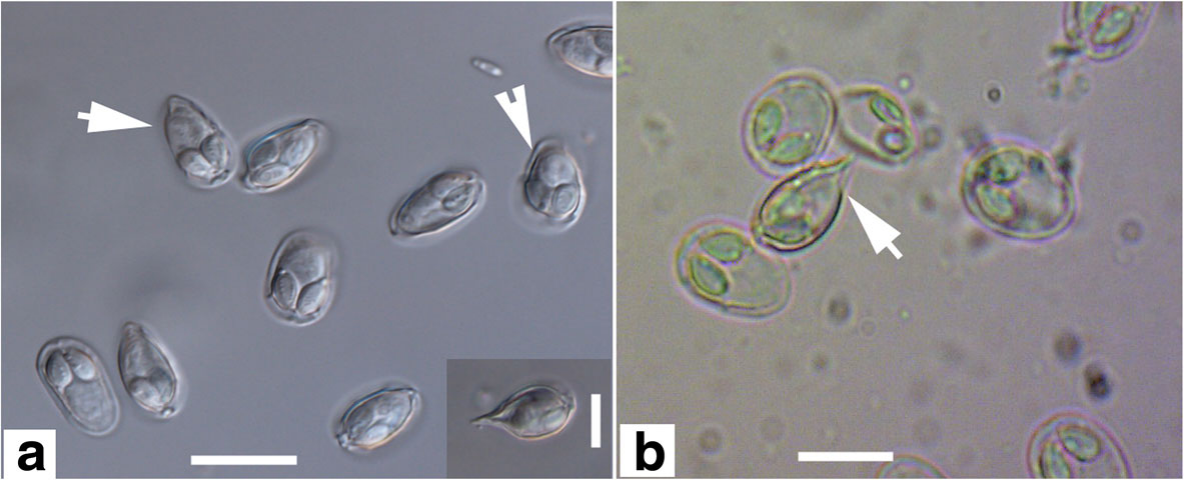
Abnormal myxospores of *Myxobolus pronini* n. sp. under light microscopy. **a** Incompletely developed abnormal spores (*arrows*). **b** *Henneguya*-like spore (*arrow*). *Scale-bars*: 10 μm

Myxospores elongated obovate in frontal view, lemon-shaped in sutural view, with blunt anterior end wider than posterior end (Figs. 2 and 3). Spore measurements as follows: Russian isolate [14.3–16.2 (15.1 ± 0.3) long, 9.6–10.8 (10.1 ± 0.1) wide, 6.4–7.4 (6.7 ± 0.15) thick]; Chinese isolate [13.8–15.6 (14.7 ± 0.24) long, 9.0–13.3 (9.6 ± 0.65) wide, 6.2–7.2 (6.6 ± 0.16) thick]. Spore valves relatively thin, symmetrical, smooth. Polar capsules 2, pyriform, equal in size, converging anteriorly, measuring as follows: Russian isolate [4.3–6.7 (5.4 ± 0.63) long, 4.8–5.6 (3.0 ± 0.16) wide]; Chinese isolate [4.8–5.6 (5.3 ± 0.16) long, 2.9–3.4 (3.0 ± 0.12) wide] (Table 2). Polar filaments coiled with 5–6 turns, situated perpendicularly to the longitudinal axis of polar capsules; 3–5 folds observed at posterior end of some spores. Intercapsular appendix and mucous envelope not observed, but a round iodinophilous vacuole remarkable.

**Table 2:** Comparative data for spore measurements (range ± standard deviation in micrometres) of *Myxobolus pronini* n. sp. and morphologically similar species

Species	Host	Site in host	SL	SW	ST	PCL	PCW	PFC	Reference
*M. pronini* n. sp. Russian isolate	*Carassius auratus gibelio*	Abdominal cavity	14.3–16.2 (15.1 ± 0.3)	9.6–10.8 (10.1 ± 0.1)	6.4–7.4 (6.7 ± 0.15)	4.3–6.7 (5.4 ± 0.63)	2.2–3.6 (3.1 ± 0.05)	5–6	This study
*M. pronini* n. sp. Chinese isolate	*C. auratus gibelio*	Mesentery	13.8–15.6 (14.7 ± 0.24)	9.0–13.3 (9.6 ± 0.65)	6.2–7.2 (6.6 ± 0.16)	4.8–5.6 (5.3 ± 0.16)	2.9–3.4 (3.0 ± 0.12)	5–6	This study
*M. ellipsoides*	*Tinca tinca*	Gills, liver, intestine	12–15	9–11		4			[9]
*M.qiankiangensis*	*C. auratus auratus*	Spleen, body cavity	15.6–18.0 (17.3)	10.6–12.0 (11.4)	8.0–8.4 (8.3)	7.8–9.6 (8.6)	3.6–4.2 (3.8)	7–9	[8]
*M. sigini*	*Hypophthalmichthys molitrix*	Body cavity	9.8–11.3 (10.6)	7.2–7.8 (7.4)	4.8–6.0 (5.3)	4.8–5.0 (4.9)	2.4–2.6 (2.5)	6–7	[11]
*M. pfrille*	*Chrosomus neogaeus*	Body cavity	12.0–14.4 (12.9)	8.4–10.2 (9.5)	(7.0)	5.0–6.0 (5.6)	3.6	5	[11]
*M. hypseleotris*	*Micropercops cinctus*	Skin, muscle, intestine	12.6–15.6 (14.6)	8.4–10.8 (9.7)	6.0–7.2 (6.6)	4.8–6.0 (5.4)	2.8–3.6 (3.3)	5–6	[11]
*M. lentisuturalis*	*C. gibelio*	Muscle	11.2–12.4 (11.8)	7.2–8.4 (7.6)	(5.2)	4.0–4.4 (4.2)	2.0–2.8 (2.5)	4	[29]

*Abbreviations*: *SL* spore length, *SW* spore width, *ST* spore thickness, *PCL* polar capsule length, *PCW* polar capsule width, *PFC* polar filament coils

In addition to the above described normal spores, there were less than 5 % abnormal spores on average in all individual plasmodia of the two geographical isolates, among which some possessed *Henneguya*-like caudal appendages (Fig. 5).

### Morphometric variation

The mature spores from the fish host in the two localities showed typical characteristics of the genus of *Myxobolus* and were very similar in shape. There was an overlap in the morphometric data although some features exhibited significant differences (Table 2, Fig. 4). The length and width of spores and the length of the polar capsules of the Russian isolates was significantly greater than those of the Chinese isolates (spore length: *t*_(98)_ = 15.25, *P* < 0.001; spore width: *t*_(78)_ = 6.462, *P* < 0.001; polar capsule length: *t*_(98)_ = -3.833, *P* < 0.001). However, the thickness of the spores and the width of the polar capsules of the two isolates were almost identical (spore thickness: *t*_(98)_ = -0.889, *P* = 0.377; polar capsule width: *t*_(98)_ = 1.460, *P* = 0.150).

### Molecular characterisation

Ribosomal RNA gene sequences of the two geographical isolates (6,068 and 6,056 bp for the Russian and Chinese isolate, respectively) were obtained after trimming the ambiguous positions in the two sequencing ends and the applied amplified primers, both with about 45.7 % G + C content. The SSU rDNA sequences of the two isolates (1,734 bp and 1,739 bp for the Russian and Chinese isolate, respectively; accession numbers KU524890 and KU524889, respectively) exhibited a similarity of 99.5 %, whereas the LSU rDNA sequences of the two isolates (3,619 bp and 3,611 bp for the Russian and Chinese isolate, respectively; accession numbers KU524892 and KU524891, respectively) showed 99.3 % similarity. All six ITS sequences (accession numbers KU524893–KU524898) were 715 bp in length, but the intra-isolate genetic distances were generally lower than inter-isolate distances (Table 3).

**Table 3:** Genetic distance (number of different positions and p-distance; below diagonal) and percent sequence similarity (%) (above diagonal) obtained from the distance matrix based on a 715 bp ITS fragment of the Russian and Chinese isolates of *Myxobolus pronini* n. sp

	Isolate^a^	1	2	3	4	5	6
1	KU524896	–	99.7	99.6	99.6	99.6	99.6
2	KU524897	2 (0.0029)	–	99.6	99.6	99.6	99.1
3	KU524898	3 (0.0043)	2 (0.0043)	–	99.4	99.4	99.0
4	KU524895	3 (0.0043)	3 (0.0043)	3 (0.0058)	–	100	99.6
5	KU524894	3 (0.0043)	3 (0.0043)	4 (0.0058)	0 (0.0000)	–	99.6
6	KU524893	6 (0.0043)	6 (0.0086)	7 (0.0101)	3 (0.0043)	3 (0.0043)	–

^a^KU524893–KU524895 isolated from Lake Taibai, China; KU524896–KU524898 isolated from the coast of Lake Baikal watershed, Russia

**Fig. 6:**
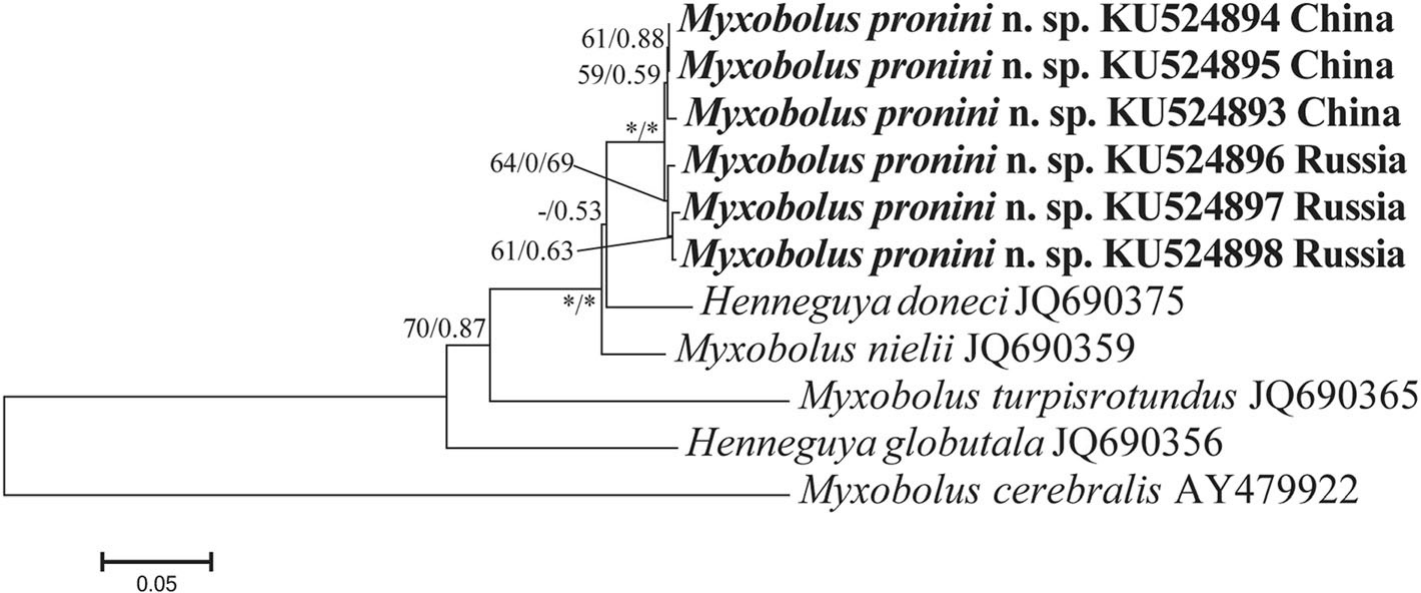
Bayesian analysis of genetic geographical differentiation of *Myxobolus pronini* n. sp. based on ITS sequences rooted with *Myxobolus cerebralis* (AY479922). Numbers at nodes indicate Bayesian posterior probability and ML bootstrap values, respectively. Asterisks indicate support values > 95 % and dashes indicate values < 50 %

**Fig. 7:**
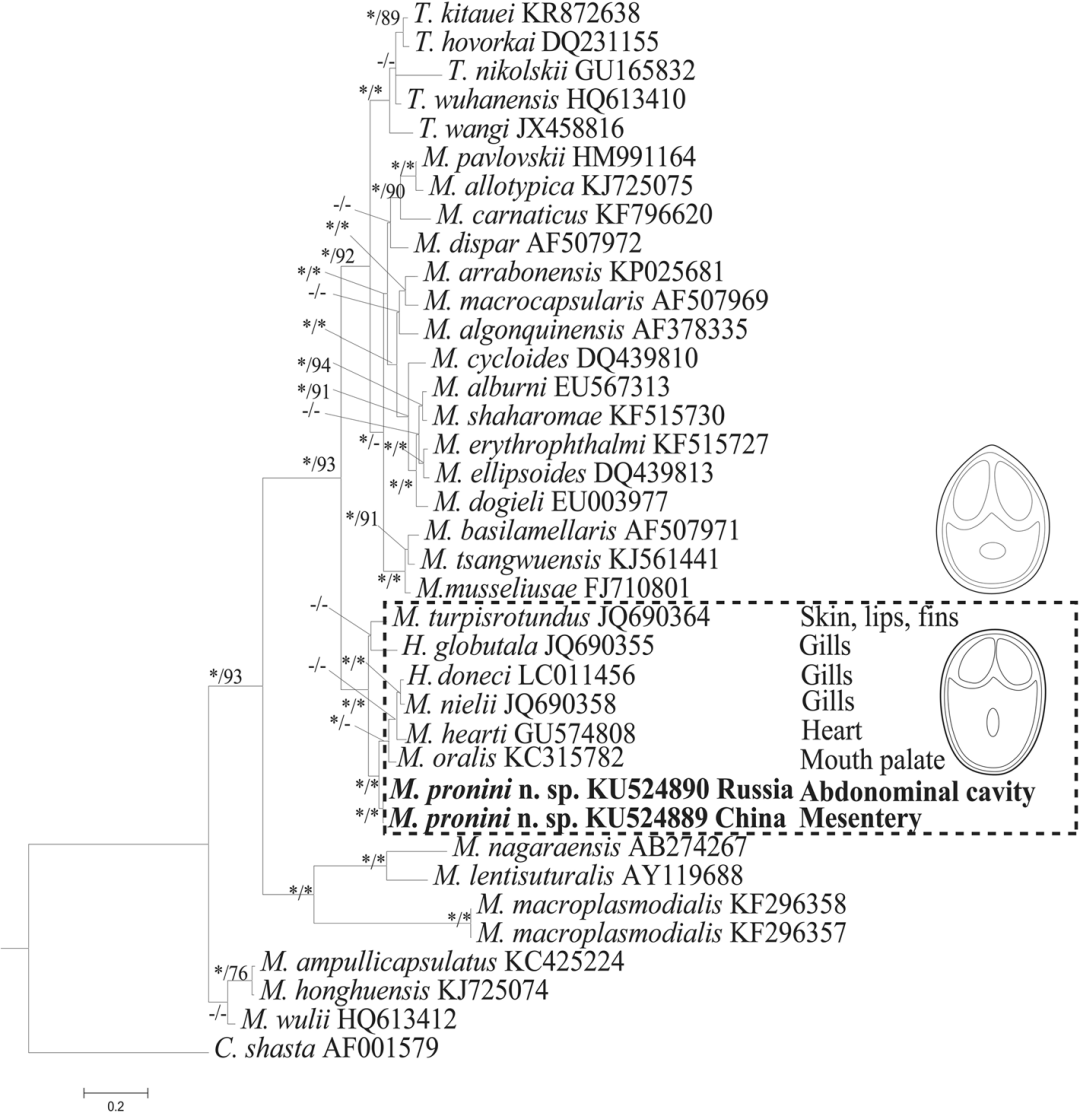
Phylogenetic tree generated by Bayesian analysis of aligned partial SSU rRNA gene sequences of *Myxobolus pronini* n. sp. and related species, rooted with *Ceranova shasta* (AF001579). Numbers at nodes indicate Bayesian posterior probability and ML bootstrap values, respectively. Asterisks indicate support values > 95 % and dashes indicate values < 50 %. The new species (indicated in bold) clustered within a cyprinid-infecting *Henneguya*-*Myxobolus* clade of parasites with round or blunt anterior ends of spores (dashed box)

Bayesian analysis of aligned full length ITS sequences revealed that *M. pronini* n. sp. forms an independent branch, clustering within the gibel carp-infecting *Henneguya-Myxobolus* clade and remarkable genetic geographical differentiation occurred for this new parasite (Fig. 6). A BLAST search using SSU rDNA of this species as queries indicated that these sequences were distinguished from all myxosporean sequences available in the GenBank database but most similar to those of *M. nielii* (Nie & Li, 1973) Landsberg & Lom, 1991 (JQ690358; 98 %), *H. doneci* Schulman, 1962 (LC011456; 98 %), *M. hearti* Chen, 1998 (GU574808; 96 %), and *H. globulata* Ye & Wang, 2012 (JQ690355; 93 %). ML and BI analyses produced similar tree topologies, although with somewhat different support values at some evolutionary nodes. Therefore, only the Bayesian tree is present here, along with the bootstrap values of ML tree. Phylogenetic analysis clearly placed *M. pronini* n. sp. in the clade of gibel carp-infecting *Myxobolus* species with round anterior end of spores under the support of high bootstrap and posterior probability value (Fig. 7).

## Discussion

Within the phylum Myxozoa, *Myxobolus* Bütschli, 1882 is the largest genus [8, 9] and the diversity keeps increasing with more attention paid by fish parasitologists over the world, especially recently from South America, Africa and India [25–27]. Out of more than 900 *Myxobolus* species reported to date, *M. pronini* n. sp. closely resembles in spore shape *M. ellipsoids* Thélohan, 1892; *M. qiankiangensis* (Chen, 1998) Eiras, Molnár & Lu, 2005; *M. sigini* (Chen, 1988) Landsberg & Lom, 1991; *M. pfrille* (Fantham, 1939) Landsberg & Lom, 1991; *M. hypseleotris* Chen & Ma, 1998; and *M. lentisuturalis* Dyková, Fiala & Nie, 2002 (Table 2). However, spores of *M. ellipsoides* are remarkably smaller than those of *M. pronini* n. sp. Additionally, *M. ellipsoides* was originally reported from gills of *Tinca tinca* in France and then recorded in most of organs of 58 distantly related fish hosts [11, 28]. So, it can be suspected that *M. ellipsoides* actually represents a cryptic species complex. Narrower anterior end of spores, longer spore length, a greater number of polar filament coils and irregular bigger plasmodium can significantly discriminate *M. qiankiangensis* from *M. pronini* n. sp., although both species were found in the abdominal cavity of *C. auratus. Myxobolus sigini* infects silver carp, *Hypophthalmichthys molitrix* Valenciennes, 1884 rather than gibel carp and can be further clearly differentiated from *M. pronini* n. sp. by the smaller size of the spores and the smaller ratio of polar capsule length to polar capsule width. *Myxobolus pfrille* and *M. hypseletris* are very similar to *M. pronini* n. sp. by having elliptical spores, but the host and spore size of these two species are different. Furthermore, a distinct triangular intercapsule appendix occurs in *M. pfrille*, but is lacking in *M. pronini* n. sp. The spore shape of *M. lentisuturalis* is very similar to that of *M. pronini* n. sp., but the two species differ in the spore size, infection site and the number of coils of the polar filament [29].

In addition to the above mentioned cypriniform-infecting species and species recorded in Russia and China, *M. macroplasmodialis* Molnár, Ranzani-Paiva, Eiras & Rodrigues, 1998, with a unique big free plasmodium (above 1 cm) in the abdominal cavity of *Salminus brasiliensis* Cuvier, 1816 (Characiformes) in Brazil [30] is morphologically similar with *M. pronini* n. sp., but the oval and antero-laterally diverged opening of the polar capsules and smaller spore size of the former undoubtedly differentiate it from the latter. Regarding the specificity of infection site of the Russian isolate of *M. pronini* n. sp., no conclusion can be made due to the lack of the early developmental stages. But, it could be presumed that plasmodia of the Russian isolate start their development in the serous membranes of the abdominal wall or visceral organs and then become detached from those sites at an advanced stage of development, similar to *M. macroplasmodialis*. Since the smaller plasmodia of the Chinese isolate were at an advanced developmental stage, determined by the presence of most of mature spores, we thought that the sporulation in the Russian isolate occurred in the serous membranes of the abdomen rather than in the visceral serous membranes and the different microhabitat produced differently sized plasmodia. Size and shape plasticity of plasmodia and spores have been widely reported for many myxosporean parasites [7, 31]. However, both isolates of *M. pronini* n. sp. represented a strict specificity to connective tissues which is consistent with most of cyprinid-infecting *Myxobolus* species [7, 32].

Simple and variable morphological features of myxospores were acknowledged to be the main factors responsible for the difficulty of myxosporean taxonomy and species identification [6, 33, 34]. As such, the two geographical isolates of *M. pronini* n. sp. showed some significant variations in the length and width of spores and the length of the polar capsules. Regional genetic differentiation of several myxozoans has been reported by phylogenetic analysis of ITS [14, 33, 35, 36]. We also demonstrated here that a distinct genetic variation occurred for two isolates of *M. pronini* n. sp. by this molecular marker. Gibel carp is widely distributed in Eurasia; its origin is still unclear, although some researchers suggest that the native habitat of the gibel carp is the Far East [37]. Due to the fact that the evolutionary migration route of the gibel carp remains unknown, the origin of speciation of this new parasite remains enigmatic. Also, some subspecies of gibel carp, including allogynogentic gibel carp have been introduced *via* anthropogenic means to wide areas for intensive culture, especially in China. Previous reports showed that pathogens from populations of cultured fish spilled-over to wild watersheds and produced genetic exchanges with wild populations and then caused genetic variations of pathogenic microorganisms [38]. However, no infection of cultured gibel carp by the new myxosporean was found despite continuing intensive investigations of the myxosporean fauna of gibel carp cultured in China during the past ten years. Therefore, factors driving this geographical genetic variation await further research.

Molecular characteristics have been widely accepted as important parameters for myxosporean taxonomy, species identification and discrimination of cryptic species [7]. Due to the lack of sufficient LSU rDNA data available on GenBank and almost congruent phylogenetic results based on LSU rDNA and SSU rDNA [17], only phylogenetic analysis of SSU rDNA sequences was performed here. The result showed that two isolates of *M. pronini* n. sp. had an unambiguously independent position within the freshwater *Myxobolus-Henneguya* clade and clustered with several gibel carp-infecting *Myxobolus* species to form an independent lineage. However, this lineage represented diverse sites of sporulation, i.e. *M. hearti* dwelling in the heart ventricle, *M. oralis* Liu, Whipps, Nie & Gu, 2014 in the palate of the mouth, *M. nielii*, *H. doneci* and *H. globulata* in the gill filaments [39] and *M. turpisrotundus* Zhang, Wang, Li & Gong, 2010 in the subepidermal tissues of the body surface, fins and gill arches [6]. So, the present result supported the viewpoint that phylogenetic affinities of the fish hosts provide stronger evolutionary signal for evolutionary relationships of histozotic myxobolids than tissue-specificity to some extent [27]. However, all species involved in this lineage possess spores with a blunt anterior end thus highlighting the importance of spore morphology as taxonomic criterion for myxosporeans. Additionally, *Myxobolus* species with caudal appendages dispersed in different groups among this lineage rather than forming an independent cluster, indicates the presence of this feature independently evolved several times from *Myxobolus* to *Henneguya* or *vice versa* [5].

Similar to other histozotic myxosporeans [11, 40], some proportion of morphological abnormal spores also occurred in most of the plasmodia of the two geographical isolates of *M. pronini* n. sp. Insufficient nutrients for normal development could not completely explain their occurrence for some abnormal spores located in the centre rather than the periphery of plasmodia. *Henneguya*-like caudal appendages of *M. pronini* n. sp. confirm once more that *Myxobolus* is closely genetically related to *Henneguya* [41], although we disagree with the suggestion of suppression of *Hennguya* by some researchers [5] to avoid further taxonomic confusion of these two speciose myxobolid groups.

## Conclusions

The two isolates infecting the serous membranes of abdominal cavity and visceral organs of the gibel carp form Russia and China are conspecific and represent a species new to science, *M. pronini* n. sp., delimitated by integrating morphological, ecological and molecular evidence. Two geographical isolates of this species represented some variations in the length and width of spores and the length of polar capsules, but within the range of intraspecific variation.

## Abbreviations

BI: Bayesian Inference analysis
ITS: Internal transcribed spacer
LSU: rDNA, large subunit ribosomal deoxyribonucleic acid
ML: Maximum Likelihood
SD: Standard deviation
SSU rDNA: Small subunit ribosomal deoxyribonucleic acid
